# Risk of Dengue Transmission in Cocody (Abidjan, Ivory Coast)

**DOI:** 10.1155/2019/4914137

**Published:** 2019-01-14

**Authors:** Diakarida Fofana, Jean Michel Vianney Beugré, Genevieve Lydie Yao-Acapovi, Sevidzem Silas Lendzele

**Affiliations:** ^1^Institut National d'Hygiène Publique (INHP) Abidjan; Ministère de la Santé et de l'Hygiène Publique (MSHP), Côte d'Ivoire; ^2^Laboratoire de Zoologie et Biologie Animale, Faculté des Sciences, Université Félix Houphouët-Boigny, Abidjan, Côte d'Ivoire; ^3^Laboratory of the Institute of Evolution and Ecology, Department of Comparative Zoology, University of Tübingen, Tübingen, Germany; ^4^Programme Onchocercoses Field Station of the University of Tübingen, Ngaoundéré, Cameroon

## Abstract

In recent years, an upsurge of vector-borne diseases has been reported in several parts of the world. Among these is dengue fever, the first arbovirus transmitted by mosquitoes of the genus* Aedes*. After the detection of the dengue virus serological types (type 1, 2, and 3) in the health district of Cocody-Bingerville in Ivory Coast, entomological investigations were carried out in the city of Cocody (host of most cases) to evaluate the risk of transmission of the disease in view of an effective vector control. Larval prospection together with the pitching of emergence traps was carried out in Caféier 5, Sideci-Coteau, Danga, Ecole de police, Gobelet village, Laurier 9, Lemania, Perles, 7^ème^ tranche, and 12^ème^ arrondissement. Entomological prospections revealed the predominance of* Aedes aegypti *(97.38%) as the main vector species of dengue viruses in Cocody. The Kruskall-Wallis test showed no statistically significant difference (KW = 1.8, p = 0.407) in the proportions of the vector species collected from the sampled sites. The risk of an outbreak of dengue fever in Cocody and other municipalities in the city of Abidjan is very certain insofar as the larval epidemic risk indices (Habitat Index, HI = 70.9; Container Index, CI = 40.26; and Breteau Index, BI= 21.3) reflect a very high epidemic risk (4 to 9) on the WHO density scale. The occurrence of* Aedes aegypti* in Cocody indicates the risk of transmission of the Dengue fever virus.

## 1. Introduction

Arboviruses (arthropod borne viruses) in recent years are in full upsurge worldwide [1, 2]. Among these arboviruses of public health importance is dengue fever. More than 390 million infections with dengue are recorded each year [3]. The disease is caused by four antigenically distinct dengue virus serotypes (DENV-1, DENV-2, DENV-3, and DENV-4). It is transmitted to humans by* Aedes* mosquitoes. According to the WHO [4], the geographical distribution of* Aedes* vectors continues to expand on a global scale. More than 128 countries are endemic to this arbovirus [5–8] and 3.97 billion people are at risk of infection [9, 10].

Three species have been described as potential vectors of dengue viruses, i.e.,* A. aegypti*,* A. albopictus*, and* A. polynesiensis*. In Africa, particularly in West Africa, recent dengue epidemics have been attributed to* A. aegypti *[11], the most active and invasive vector in the tropics [12, 13]. In Ivory Coast, several entomological prospections carried out in the city of Abidjan (Yopougon, Treichville, Marcory, Koumassi, Port-Bouet, etc.) have revealed the predominance of* A. aegypti *[14–17], a common vector of Zika, Chikungunya (CHIKV), yellow fever, and dengue (DENV) viruses. The increasing numbers of these vectors in urban areas pose a potential threat of an arbovirus epidemic. The Ministry of Public Health and Public Hygiene in 2017 revealed 481 suspected cases of dengue including two deaths reported in the health district of Cocody-Bingerville. Of these cases, 36 patients were diagnosed positive to dengue type 3, 102 dengue type 2, and nine dengue type 1. The prevailing situation triggers the following research questions: What is the risk of dengue transmission in Cocody? And what is the site with the most cases? The main goal of the present study is to assess the level of risk of dengue transmission through entomological prospections in preparedness for an efficient vector control. It will be necessary to identify the different breeding sites and potential vectors of dengue fever in the city of Cocody and to determine the different epidemic risk indices.

## 2. Material and Methods

### 2.1. Study Area

**Figure 1:**
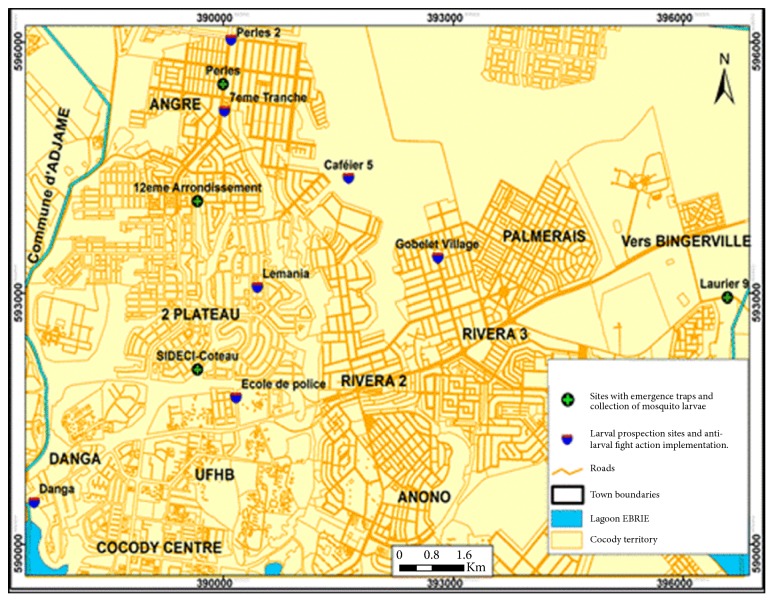
Location of the city of Cocody showing study sites.

The city of Abidjan is found along the continental shelf, north of the lagoon Ebrié. The town of Cocody is located between Latitude 5° 20′ 56^″^ N and Longitude 4° 00′ 42^″^ W (Figure 1) with an estimated area of 7,745 ha, 77.45 Km^2^, with about 447,055 inhabitants and a population density of 5.772 inhabitants/Km^2^ [18]. Based on the epidemiological and virological data of the first dengue cases reported in Cocody, different sites were targeted for the present study: notably, Caféier 5, Sideci-Coteau, Danga, Ecole de police, Gobelet village, Laurier 9, Lemania, Perles, 7^ème^ tranche, and 12^ème^ arrondissement.

### 2.2. Statistical Analysis

Data was analysed using SPSS version 21. The Kruskall-Wallis test was used to compare the proportions of* A. aegypti* obtained at the sampling sites. The Freidman test was used to compare the positivity of the different breeding sites. The statistical tests were kept at 5% probability level with a 95% confidence interval (CI).

### 2.3. Entomological Prospections

Entomological prospections were carried out in the town of Cocody from the 3^rd^ of June to the 16^th^ of August 2017 in 10 sites. The goal of this present study was to collect information on the different vectors of arboviruses and determine the different larval indices of epidemic risk in Cocody. To achieve this goal, larval prospection and pitching of emergence traps were the entomological collection methods used. The larval surveys consisted of searching for the breeding sites in and around homesteads (old tires, water storage containers, abandoned containers, leafy plants, flower vases, etc.) on materials which are likely to contain* Aedes* larvae. Mosquito larvae were collected from positive sites and brought to the insectarium of the National Institute of Public Hygiene (INHP), for breeding. As for the breeding substrate, they were removed five days after emergence and the basements dried for ten days. They underwent three consecutive watering at intervals of five days. The resulting larvae were reared to adult mosquitoes. Adult mosquitoes were identified up to the genus and species level under a binocular dissecting microscope, using the identification key of Edwards [19] and Huang [20].

## 3. Results

### 3.1. Culicids Fauna of Cocody

Entomological prospection led to the identification of five species of mosquito:* A. aegypti*,* Anopheles gambiae*,* Culex annulioris*,* C. cinereus*, and* C. quinquefasciatus* (Table 1). In all prospected sites,* A. aegypti *(97.38%) was the most frequent vector species of arboviruses. At the homestead-level,* A. aegypti* occurrence was as follows: Laurier 9 (96.12%) and Pearls (97.37%), while in the sites of Sideci-Coteau and 12^ème^ arrondissement, the frequency of this species was 100%. The Kruskal-Wallis test did not show any statistically significant difference in the proportions of* A. aegypti* in the different sites (KW = 1.8, p>0.001).

### 3.2. Mosquito Breeding Sites in Cocody

A total of 1053 breeding sites were found in the town of Cocody during larval surveys. The largest number of positive breeding sites was observed at 7^ème^ tranche with 344 potential breeding sites and the lowest number encountered at Caféier 5. However, the highest number of positive breeding sites was observed at the Ecole de police (73, 55%) followed by Goblet village (56.84%), Danga (48.95%), Caféier 5 (44.87%), Perles (32.4%), 7^ème^ tranche (28.19%), and Lemania (24.43%) (Table 2). The points harboring the larvae of the genus* Aedes* (40.26%) were categorized into five groups, i.e., water storage containers, abandoned containers, tyres, natural habitats, and other dwellings. Of these, abandoned containers (133, or 31.3%) were heavily infested, followed by water storage containers (26.17%), tyres (24.17%), other deposits (10.85%), and natural biotopes (7.07%) (Table 2). The Freidman test showed a highly significant difference between the positive sites observed in the different sites (F=16.133, p≤0.001). This positivity differed with the nature of the breeding site encountered (F = 17.853, p≤0.001).

### 3.3. Larval Epidemic Risk Indices

A total of 189 sites were sampled during the larval surveys in the town of Cocody, i.e., Caféier 5 (n=25), Danga (n=25), Ecole de police (n=36), Goblet village (n=23), Lemania (n=10), Perles (n=37), and 7^ème^ tranche (n=33). The larval epidemic risk index was for all surveyed sites and differed with sampled substrate as follows: Habitat Index (HI, 70.90), Container Index (CI, 40.26), and Breteau Index (BI, 21.30) threshold level range of 4-9 (Table 3). These indices reflect a high epidemic risk on the WHO [21] density scale.

## 4. Discussion

The present entomological prospections in the town of Cocody led to the identification of five species of culicids, i.e.,* A. aegypti*,* Anopheles gambiae*,* C. annulioris*,* C. cinereus*, and* C. quinquefasciatus*.* A. aegypti *(97.38%) was the main vector species of arboviruses in Ivory Coast. This species is mostly found in most municipalities of the city of Abidjan (Marcory, Treichville, Port-Bouet, Koumassi, Yopougon, etc.) as earlier reported by Guindo-Coulibaly [14]; Konan et al. [16]; Coulibaly [17]. It is capable of adapting to all living environments, especially in urban areas due to variations in climate and temperature [22–25] as well as certain factors related to molecular, genetic (intrinsic) factors and the density of the vector (extrinsic factor) which affects the vector's competence and the chances of getting in contact with humans [26].

The predominant role of humans in the proliferation of arbovirus vectors is well-known. Indeed, it is clear from some literature [4, 27, 28] that most human activities (uncontrolled urbanization and population exodus) and the high cost of living are at the origin of proliferation of mosquito breeding sites. In Ivory Coast according to Koné et al. [15], the abundance of substrates that favors the development of* A. aegypti* in Abidjan is justified largely by the massive influx of population from other towns to Abidjan during the 2002 crisis, deterioration of the living environment, and circulation of the virus in mosquito-vectors. It is also necessary to add ignorance of the population as determining factor in larval breeding [29]. In Cocody, the preferential breeding sites of* A.* were abandoned containers followed by water storage containers and tyres. Main seasonal deposits [14, 30] and abandoned containers showed the highest number of breeding sites (31.3%). In a study previously conducted in this same municipality [17], the storage containers resulted in the highest breeding sites. The season (rainy season) at which this study was conducted could justify the results obtained. Also, 7.7% of the natural deposits were positive to* A. *larvae. This proportion shows that these deposits can be used for breeding by the* Aedes*, especially because of the duration and availability of the water contained under sheds of plants. In Cocody, the preferential* A. aegypti* breeding grounds were grouped according to their nature: abandoned containers, water storage containers, and tyres. Seasonal deposits [14, 30] and abandoned containers (domestic waste) were highly infested (31.3%). In a study previously conducted in this same area by Coulibaly [17], storage containers were most common breeding sites for* Aedes*.

The presence of vectors (particularly* A. aegypti*) and the abundance of their breeding sites as well as the occurrence of different larval epidemic risk indices (HI = 70.9, CI = 40.26 and BI = 21.3) project a high epidemic risk of the order of 4-9 on the WHO [21] density scale. All these values reflect the existence of* A. aegypti* density enough to cause and maintain an outbreak of dengue fever. These values obtained were largely influenced by the refusal of the populations of the different study sites to cooperate. One of the reasons given was the insecurity caused by the postelection crisis of 2010. Nevertheless, studies previously conducted in Cocody by Coulibaly [17] showed a high epidemic risk of arboviruses (Chikungunya). The risk value was evaluated and reported as six on the WHO density scale.

## 5. Conclusion

The entomological prospections conducted in the town of Cocody resulted in the identification of* Aedes aegypti*, vector species of the dengue virus.* A. aegypti *is responsible for most of the yellow fever epidemics, Chikungunya, and Zika in Ivory Coast and is practically found in all the communities of the city of Abidjan. Unfortunately, the behavior of the population contributes to the increase density of this vector and its maintenance in the urban environments which harbor the various potential breeding sites (abandoned containers, water storage containers, tyres, etc.) that favor their proliferation. The determined larval epidemic risk indices showed that the risk of a dengue epidemic occurring in the town of Cocody is very high (4 to 9) when comparing the index ranges obtained in the study to that on the WHO density scale. To prevent the occurrence and spread of dengue or other arboviruses, vector control remains the only possible outcome. This requires that all possible measures be taken to destroy the mosquitoes likely to harbor the virus and to prevent the new production of mosquitoes via the management of breeding substrates that favors their proliferation.

## Figures and Tables

**Table 1:** Culicids fauna collected from different sampled sites.

Collection method	Species identified	Laurier 9	Perles	Sideci-Coteau	12^ème^ arrondissement
	*Aedes aegypti ∗*	64 (100%)	159 (94.64%)	312 (100%)	442 (96.71%)
	*Anopheles gambiae*	0 (0.00%)	0 (0.00%)	0 (0.00%)	10 (2.19%)
Larval prospection	*Culex annulioris*	0 (0.00%)	0 (0.00%)	0 (0.00%)	1 (0.22%)
	*Culex cinereus*	0 (0.00%)	0 (0.00%)	0 (0.00%)	4 (0.88%)
	*Culex quinquefasciatus*	0 (0.00%)	9 (5.36%)	0 (0.00%)	0 (0.00%)
	Total prospection	64	168	312	457
Emergence trap	*Aedes aegypti ∗*	40 (100%)	64 (100%)	280 (100%)	114 (100%)
Total		104	232	592	571

*∗* means vector of dengue.

**Table 2:** Proportion and characteristics of *Aedes *breeding sites in the prospected areas.

Sites	Number	Positive breeding sites	water storage containers (WSC)	Abandoned Containers (AC)	Tyres	Natural breeding grounds (NBG)	Other breeding sites (OBS)
Caféier 5	78	36 (46.15%)	12 (33.33%)	5 (13.89%)	11 (30.56%)	1 (2.78%)	7 (19.44%)
Danga	143	68 (47.55%)	7 (10.29%)	15 (22.06%)	35 (51.47%)	7 (10.29%)	4 (5.88%)
Ecole de police	121	89 (73.55%)	14 (15.73%)	58 (65.17%)	6 (6.74%)	0 (0.00%)	11 (12.36%)
Gobelet village	95	54 (56.84%)	29 (53.70%)	7 (12.96%)	6 (11.11%)	12 (22.22%)	0 (0.00%)
Lemania	164	45 (27.43%)	15 (33.33%)	7 (15.56%)	17 (37.78%)	0 (0.00%)	5 (11.11%)
Perles	108	35 (32.40%)	12 (34.29%)	8 (22.86%)	2 (5.71%)	8 (22.86%)	5 (14.29%)
7^ème^ tranche	344	97 (28.19%)	22 (22.68%)	33 (34.02%)	26 (26.80%)	2 (2.06%)	14 (14.43%)
Total	1053	424 (40.27%)	111 (26.18%)	133 (31.37%)	103 (24.29%)	30 (7.08%)	46 (10.85%)

WSC: barrel, basin, 20 L tin, tank, bowl, bucket, kettle, fountain, barrel.

AC: disposable plate, disposable cup, paint bucket, bidet, bottle, canary, toilet tank, wash basin, sales cart, cooler, fridge frame, box, stove, hairdressing equipment.

NBG: puddle, banana, tree hollow, snail shell, coconut shell, stagnant.

OBS: under flower pots, plastic chair, floor siphon, sodeci lid, trash bucket lid, paint bucket lid, flower pot, cover hollows, pool, gutter, sheet metal hollows.

**Table 3:** Epidemic risk indices of the different surveyed sites.

Prospection sites	Number of Sampled sites	Potential breeding sites	Positive breeding sites	Habitat Index (HI)	Container Index (CI)	Breteau Index (BI)	WHO Scale
Caféier	25	78	36	62.96	46.15	184.60	8-9
Danga	25	142	68	88.00	47.55	190.20	8-9
Ecole de police	36	121	89	54.05	73.55	204.30	7-9
Gobelet village	23	95	54	86.95	56.84	247.13	9
Lemania	10	164	45	72.72	27.43	274.30	7-9
Perles	37	108	35	48.38	32.40	87.56	7-8
7^ème^ tranche	33	344	97	94.11	28.19	5.42	7-9
Total	189	1 053	424	70.90	40.26	21.30	4-9

## Data Availability

The survey data on transmission risk of dengue fever virus are included within the article. The data used to support the findings of this study are available from the corresponding author upon request.
